# Genome-wide signatures of selection and genetic diversity reveal the impact of modern breeding on Brazilian soybean

**DOI:** 10.1007/s11032-026-01679-0

**Published:** 2026-05-28

**Authors:** Brizza Vargas, Newton Deniz Piovesan, Cleberson Ribeiro, Maximiller Dal-Bianco

**Affiliations:** 1 Laboratório de Bioquímica Genética de Plantas, 212, BIOAGRO, Universidade Federal de Viçosa, Viçosa, MG 36570-900 Brazil; 2 Departamento de Biologia Geral, Universidade Federal de Viçosa, Viçosa, MG 36570-900 Brazil; 3 Departamento de Bioquímica E Biologia Molecular, Universidade Federal de Viçosa, Viçosa, MG 36570-900 Brazil

**Keywords:** Glycine max, Genetic diversity, Population structure, Selection signatures, Soybean breeding

## Abstract

Soybean (Glycine max (L.) Merr.) breeding in Brazil has driven remarkable gains in yield and adaptation; however, intensive selection raises concerns regarding the erosion of genetic diversity in modern cultivars. Here, we conducted a genome-wide analysis of genetic diversity, population structure, linkage disequilibrium, and selection signatures in Brazilian soybean germplasm using high-density SNP genotyping. Ninety-five genotypes from the Soybean Breeding Program for Quality Traits at UFV, including commercial cultivars and advanced breeding lines from both in-house and external sources, representing different breeding periods were genotyped with the 180K Axiom® Soybean SNP array, and 61,712 high-quality SNPs were retained after quality control. Population structure analyses consistently identified two main genetic groups largely corresponding to older and modern cultivars, along with an admixed group. Modern cultivars exhibited reduced nucleotide diversity and lower Tajima’s D values compared with older genotypes, while AMOVA, Fst, and Nei genetic distance estimates indicated moderate genetic differentiation between populations. Genome-wide linkage disequilibrium was extensive, with slow LD decay, reflecting the effects of self-pollination, domestication bottlenecks, and strong directional selection. BayeScan analysis detected 581 SNPs exhibiting signatures of positive directional selection (α > 0), indicating elevated genetic differentiation between groups, many of which overlapped with previously reported QTLs related to yield components, seed composition, and plant architecture. A pronounced selection hotspot on chromosome 19 showed near-complete haplotype differentiation between older and modern cultivars, highlighting a genomic region repeatedly targeted during recent breeding cycles. Together, these results demonstrate that contemporary soybean breeding in Brazil has reshaped genome-wide diversity by intensifying selection at key loci while maintaining a narrow genetic base, providing important insights for future breeding strategies aimed at sustaining long-term genetic gain.

**Supplementary Information:**

The online version contains supplementary material available at 10.1007/s11032-026-01679-0.

## Introduction

Soybeans (Glycine max (L.) Merr.) are a legume used for over 5,000 years in human food and, more recently, as a base for animal feed and biodiesel production (Vello and Silva 2006). In Brazil, the crop has consolidated as the main pillar of modern agro-industrialism, making the country the world's largest producer since 2021 (Sal 2025). Brazil harvested 171.5 million tons of grains in 2024/2025, representing a 13% increase compared to the 2023/2024 harvest, in a cultivated area of 47,350.6 thousand hectares. In the global scenario, Brazil accounted for 40% of total production, followed by the United States with 28% (CONAB 2025; USDA, 2025).

The history of soybean cultivation in Brazil is relatively recent. Although early commercial records in the southern state of Rio Grande do Sul date back to the 1930 s, large-scale expansion began in the 1960 s, heavily supported by the successful adaptation of cultivars introduced from the southern United States (Bonato and Bonato 2002). As the crop's economic importance grew, breeders began intensely crossbreeding these introductions to develop national varieties. A major milestone in this process was the incorporation of the long juvenile period trait, which allowed the development of cultivars adapted to lower latitudes and drove the massive expansion of soybean into the Brazilian Cerrado (Priolli et al. 2004; Silva et al. 2025). Despite this successful geographic adaptation, studies consistently demonstrate that the genetic base of Brazilian soybean remains remarkably narrow (Hiromoto and Vello 1986; Silva et al. 2024; Maldonado dos Santos et al. 2022). Specifically, just four ancestors (CNS, S-100, Roanoke, and Tokyo) account for more than half of the total genetic contribution to the Brazilian germplasm (Wysmierski and Vello 2013). More recently, the widespread adoption of crop succession systems (such as double-cropping) has driven breeding programs to focus intensely on the accelerated development of early-maturing cultivars, a modern shift that continues to pressure and squeeze this genetic bottleneck (Gwinner et al. 2017).

Analyses using molecular markers, such as SSRs and SNPs, reveal low genetic variability and high similarity among genotypes, reflecting intense selection for agronomic traits of interest, including flowering time, maturation, pathogen resistance, water use efficiency, and tolerance to nutritional deficiencies (Gwinner et al. 2017; Mendonça et al. 2022). This selective pressure has resulted in allele fixation, reduced genetic diversity, and selective sweeps in genomic regions associated with QTLs important for productivity and adaptation (Maldonado dos Santos et al. 2022; Silva et al. 2024, 2025), demonstrating that Brazilian breeding programs have strongly shaped the genomic composition of local cultivars.

Although widely used, the definition of genetic diversity is broad and may lead to distinct interpretations, encompassing variation from the nucleotide level to the entire genome of individuals and populations (Fu 2015). Genetic diversity is essential for maintaining healthy populations, as different genes associated with resistance and other traits can act in a complementary manner. Conversely, low genetic variability increases vulnerability to pests, environmental stresses, and climate change (Salgotra and Chauhan 2023). The pursuit of sustainable agriculture requires balancing productivity gains with the preservation of crop genetic diversity. Achieving this balance depends on understanding the impacts of breeding strategies on genetic variability, allowing the continuous development of superior genotypes without significant losses of diversity (Fu 2015).

Due to the economic importance of soybeans, genetic breeding programs have prioritized high yield, pest resistance, and earliness. In Brazil, particularly following the expansion of off-season corn cultivation, the demand for early-maturing cultivars has intensified the crop’s genetic bottleneck (Gwinner et al. 2017). Selection for these traits generates strong selective pressure in associated genomic regions, where high levels of linkage disequilibrium can be observed. Therefore, molecular characterization of the germplasm used is fundamental for the development of new cultivars (Voss-Fels and Snowdon 2016).

With advances in next-generation sequencing (NGS) technologies, it has become possible to analyze entire genomes and identify millions of SNP markers (Valliyodan et al. 2016). These innovations have enabled the development of high-throughput genotyping techniques that are widely used in genetic diversity studies at relatively low cost. Several genotyping methods have been developed, including SoySNP50K, 180fK AXIOM® SoyaSNP, and BARCSoySNP6K (Lee et al. 2015; Song et al. 2013, 2020; Silva et al. 2025). According to Fischer et al. (2017), SNPs derived from NGS technologies are particularly suitable for studies aimed at inferring and comparing genetic diversity within and between populations and species, as sequencing-based genotyping approaches have become robust and economically accessible.

Although extensive genomic resources are available for soybean, most studies focusing on Brazilian germplasm are biased toward older cultivars and fail to represent the modern materials that currently dominate soybean production in Brazil. Over the last two decades, soybean breeding in the country has been strongly influenced by the expansion of off-season maize cultivation, driving the selection of earlier maturing and highly productive cultivars capable of optimizing land use (Gwinner et al. 2017). While the agronomic and breeding aspects of this process have been previously described (Bigolin and Talamini 2024), comprehensive genome-wide data for contemporary Brazilian cultivars remain scarce. Here, we analyzed 95 soybean genotypes spanning different breeding periods, with an emphasis on modern cultivars, to characterize genetic diversity, population structure, and genomic regions under selection in Brazilian soybean. By integrating high-density SNP genotyping with population genomic analyses, this study provides new insights into the genomic consequences of recent breeding practices and delivers valuable information to support future soybean improvement strategies under current Brazilian agricultural systems.

## Material and methods

### Plant material

A total of 96 soybean genotypes were initially selected from the Soybean Breeding Program for Quality Traits germplasm collection at the Universidade Federal de Viçosa (UFV), which comprises materials developed within the program as well as genotypes originally sourced from private companies and public institutions (Supplemental Table S1). This panel encompasses both widespread commercial cultivars and advanced breeding lines belonging to our program. To ensure a comprehensive representation of modern and historical Brazilian germplasm, genotypes were specifically chosen to maximize diversity in distinct phenotypic characteristics, such as maturity group and regions of adaptation in Brazil. The selection captured a wide temporal diversity and represent different genetic backgrounds, since the selected materials include cultivars developed by different breeding companies (Supplemental Table S1).

### Genotyping

The 96 genotypes were grown in a greenhouse at the Universidade Federal de Viçosa, and leaf discs were collected and stored at − 80 °C. Genomic DNA was extracted following the protocol described by Dellaporta et al. (1983). DNA quality was assessed by electrophoresis on a 0.8% agarose gel, and DNA concentration was determined using a NanoDrop spectrophotometer (NanoDrop Technologies, Wilmington, DE, USA). Samples were diluted to a final concentration of 50 ng μL⁻^1^ and lyophilized in a 96-well plate.

Genotyping was performed by Eurofins BioDiagnostics LLC (River Falls, WI, USA) using the 180K Axiom® Soybean Genotyping Array (Thermo Scientific), which comprises 180,961 polymorphic SNP markers distributed across the 20 soybean chromosomes. The markers are polymorphic in both wild and cultivated soybean accessions (Lee et al. 2015). The analyses were based on the Glycine max Wm82.a2 reference genome assembly. No coordinate conversions were performed, and this assembly version was used for all downstream genomic and QTL analyses. SNP markers were filtered according to the manufacturer’s recommendations: Dish Quality Control (DQC) ≥ 0.82, QC call rate ≥ 97%, average call rate for approval ≥ 98.5%, and minor allele cutoff ≥ 2. Data quality control was conducted using the Axiom™ Analysis Suite software following the manufacturer’s guidelines.

### Data filtering

Additional filtering criteria were applied to refine the dataset, retaining markers with ≤ 40% missing data, minor allele frequency (MAF) ≥ 0.05, and ≤ 20% heterozygosity. During this quality control step, one genotype was excluded due to poor data quality. Consequently, a final panel of 95 genotypes and 61,712 SNP markers was retained for all downstream analyses.

### Population structure

Population structure was inferred using STRUCTURE software (v.2.3.4) (Pritchard et al. 2000) based on a Bayesian clustering approach, using the 61,712 filtered SNPs. Five independent runs were performed under a no-admixture model with correlated allele frequencies. Each run consisted of a burn-in period of 100,000 iterations followed by 100,000 Markov chain Monte Carlo (MCMC) iterations.

The number of hypothetical subpopulations (K) ranged from 1 to 10, and the most likely K value was determined using the ΔK method (Evanno et al. 2005) implemented in the Structure Selector online platform. Population assignment was based on the membership coefficient (Q) estimated by STRUCTURE. Individuals with Q ≥ 0.8 were assigned to one of the two main populations (Q1 and Q2). Genotypes with Q values < 0.8 for both populations were grouped into a third population (A), representing individuals with intermediate genetic composition. The classification in two main genetic groups, largely corresponding to older (Q1) and modern cultivars (Q2), along with an admixed group (A) emerged strictly *a posteriori* from the STRUCTURE analysis when clustering results were interpreted alongside genotype release dates.

To account for marker redundancy, the initial set of 61,712 SNPs was subjected to linkage disequilibrium (LD) pruning using PLINK software (Purcell et al. 2007), resulting in a subset of 2,334 independent SNPs. Principal component analysis (PCA) was then performed on this pruned dataset using TASSEL software (v.5.0) (Bradbury et al. 2007), considering the first five principal components. Clustering analysis was conducted in R (v.4.4.1) (R Core Team 2024) using the k-means algorithm, with the optimal number of clusters determined by the silhouette method. The scores of the first two principal components were used to generate graphical representations in R using the ggplot2 package (Wickham 2016).

### Linkage disequilibrium

Linkage disequilibrium (LD) was estimated for all chromosomes using TASSEL software (v.5.0) with a sliding window of 50 SNPs. LD analysis was also performed separately for each chromosome. LD values were considered significant at *p* < 0.01. The average LD decay rate was estimated as the physical distance at which the r^2^ value decreased to half of its maximum. Visualization of LD decay patterns was performed using R (v.4.4.1).

### Genetic distance and relatedness

A genomic relatedness matrix was generated in TASSEL using the centered identity-by-state (IBS) method (Endelman and Jannink 2012). Genetic distance matrices were also calculated and used to construct a dendrogram based on the Neighbor-Joining method. Graphical visualizations were produced in R using the ape (Paradis et al. 2004) and ggtree (Yu et al. 2017) packages. To facilitate interpretation, tree tips were colored according to the population assignments inferred by STRUCTURE.

## Genetic diversity analysis

Nucleotide diversity (*π*) and heterozygosity percentages were estimated using TASSEL software (Bradbury et al. 2007). Additional genetic diversity analyses, including analysis of molecular variance (AMOVA) (Excoffier et al. 1992) and Nei’s genetic distance (Nei 1978), were conducted using the adegenet (Jombart 2008) and poppr (Kamvar et al. 2014) packages in R.

### Selection analysis

To identify genomic regions under selection and identify genomic regions impacted by breeding, we employed an ensemble approach compiling multiple selection indices. The locus-specific fixation index (*FST*) was calculated using PLINK software (Purcell et al. 2007), and Tajima's D neutrality test (Tajima 1989) was estimated using TASSEL software (Bradbury et al. 2007) to provide a complementary overview of selection signatures across the genome. In parallel, candidate loci exhibiting signatures of selection (as defined by positive α values in BayeScan) were identified using BayeScan v.2.1 (Foll and Gaggiotti 2008), considering the two main populations (Q1 and Q2) defined by the population structure analysis.

Prior odds were set to 10. BayeScan output files were analyzed in R, and SNPs were filtered using a q-value threshold of ≤ 0.01, corresponding to a false discovery rate (FDR) of 1%. SNPs with α ≥ 0, indicative of positive directional selection, were considered candidate loci under selection. Visualization of BayeScan results was performed using the ggplot2 package (Wickham 2016).

Furthermore, SNPs identified as candidates for positive directional selection (α > 0, as defined by BayeScan) were analyzed to define haplotype blocks using Haploview software (Barrett et al. 2005), applying the Solid Spine of LD criterion. The corresponding genomic regions were examined in SoyBase to identify previously reported QTLs located within each haplotype block. The comprehensive distribution of selected SNPs, selection indices (BayeScan and *FST*), and associated QTLs along the soybean chromosomes was visualized collectively using a Circos plot.

## Results

### Genome-wide SNP distribution

After quality filtering, a total of 61,712 high-quality polymorphic SNP markers distributed across the 20 soybean chromosomes were retained for downstream analyses, representing approximately 34.1% of the total markers present on the array. We excluded CD213PTA genotype because it did not meet the defined filtering parameters. We obtained high number of markers distributed across the twenty chromosomes (Supplemental Fig. 1), with an average of 3085 markers per chromosome, ranging from 2,089 (chromosome 12) to 4,539 (chromosome 18).

### Genetic similarity and diversity among genotypes

The genetic distance matrix (Endelman and Jannink 2012) revealed high genetic similarity among Brazilian soybean genotypes (Supplemental Table S2). Despite representing different breeding programs and release years, the average distance found between the 95 genotypes in pairs was 0.33, reflecting limited genome-wide divergence. (Supplemental Fig. 2).

### Population structure and clustering analyses

**Fig. 1:**
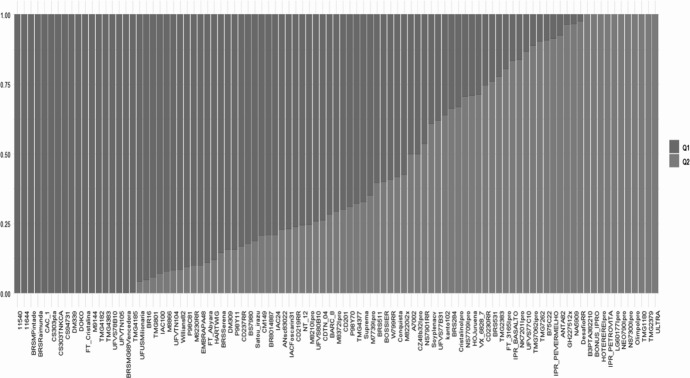
Population structure of 95 soybean genotypes inferred using STRUCTURE (K = 2). Each vertical bar represents an individual genotype, and the y-axis indicates the membership coefficient (Q). Genotypes are grouped according to their genetic ancestry proportions. The colors represent the two inferred genetic subpopulations and admixed individuals: subpopulation 1 (Q1, ancestral/older population) is shown in salmon, and subpopulation 2 (Q2, modern population) in cyan. Individuals were assigned to a population based on a membership coefficient threshold of Q ≥ 0.80

**Fig. 2:**
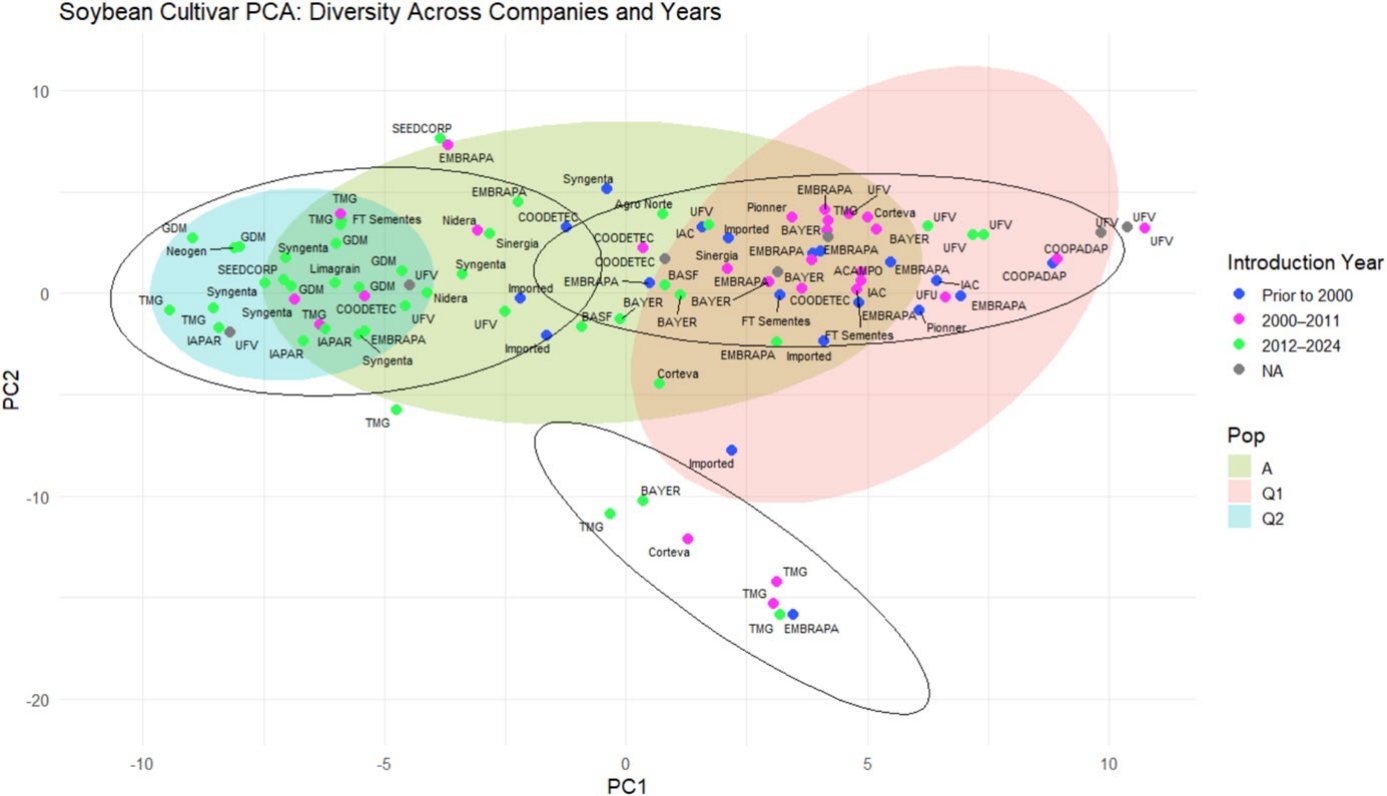
Principal component analysis (PCA). The first two principal components (PC1 and PC2) explained 7.08% and 5.31% of the total variation, respectively. The plot reveals three clusters, delineated by black ellipses, with cluster 1 located on the right, cluster 2 on the left, and cluster 3 in the lower region. Points are colored according to the release period of the genotypes, as indicated in the legend, and genotypes are identified by the companies responsible for their development. Populations are defined as Q1 (ancestral/older), Q2 (modern), and A (admixed) based on STRUCTURE results

**Fig. 3:**
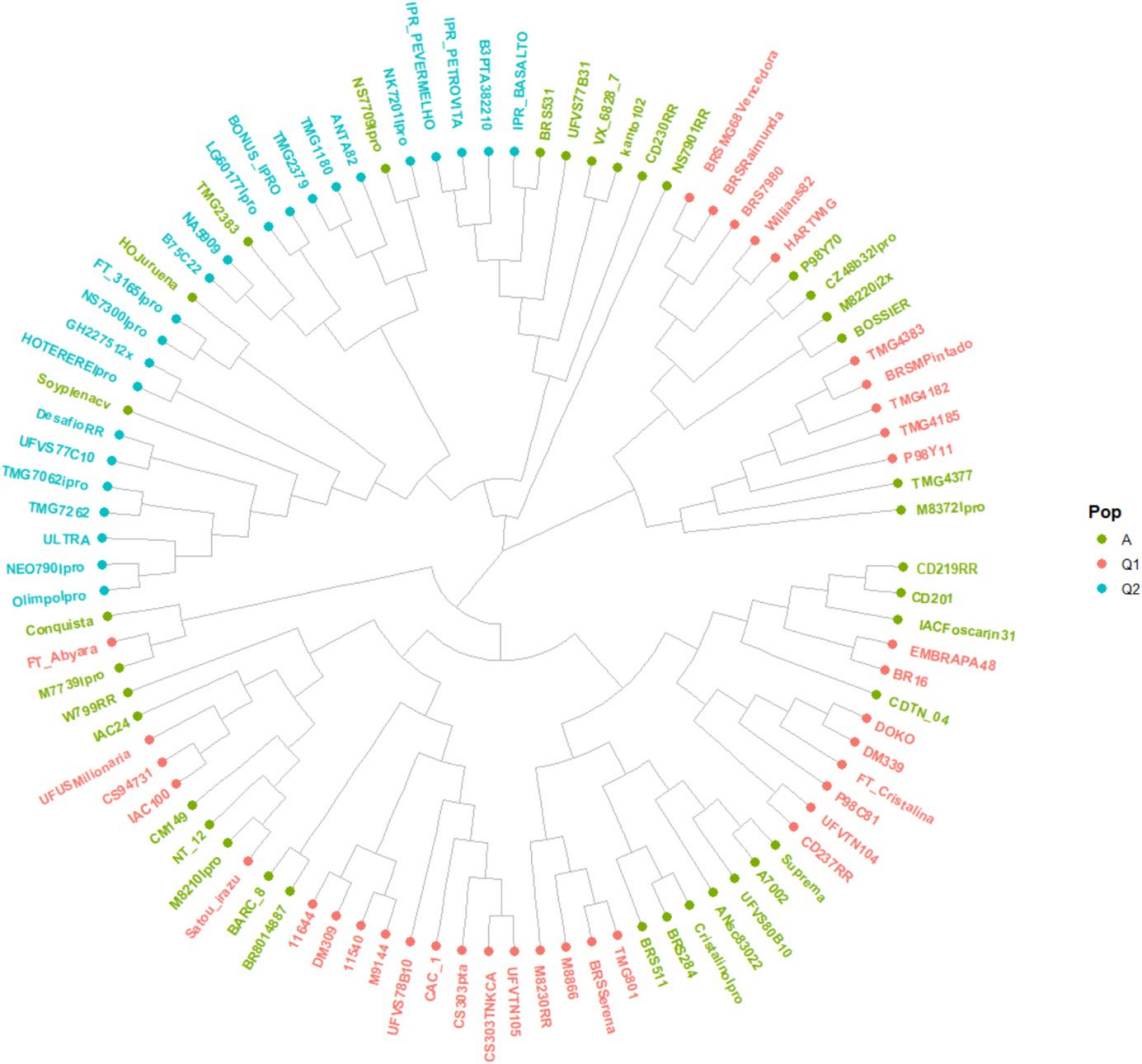
Dendrogram based on genetic distance. The dendrogram was constructed using genetic distance matrices and the Neighbor-Joining method. Branch colors correspond to the genetic groups identified by STRUCTURE (K = 2): Q1 (ancestral/older), Q2 (modern), and A (admixed)

Bayesian clustering analysis identified two major populations (Q1 and Q2) (Fig. 1). Using an affiliation rate coefficient threshold of Q ≥ 0.80 (Supplemental Table S1), individuals were confidently assigned to the two main populations (Q1 and Q2), while those with intermediate ancestry were classified as admixed (A). This population subdivision was strongly supported by principal component analysis (PCA) (Fig. 2) and Neighbor-Joining dendrograms (Fig. 3). The PCA clearly separated Q1 and Q2 along the first principal components while Neighbor-Joining dendrograms clearly separate population Q1 and Q2, with population A more disperse along the groups.

### Temporal trends in cultivar release

A clear temporal pattern was observed across populations (Supplemental Table S1). Population Q1 predominantly comprised older cultivars, while Population Q2 consisted mainly of modern cultivars, released more recently. We also observed that the number of genotypes in Q1 diminishes over the years, while Q2 increases (Supplemental Fig. 3). The admixed population showed intermediate release years. These results indicate a progressive temporal shift in the genetic composition of Brazilian soybean cultivars.

### Genetic differentiation and diversity indices

Analysis of molecular variance (AMOVA) revealed significant genetic differentiation among populations (Supplemental Table S3). Population Q1 exhibited higher nucleotide diversity (π) and Tajima’s D values than Q2 (Supplemental Table S4), whereas Q2 showed reduced genetic diversity, consistent with stronger selection pressure. Pairwise Fst and Nei genetic distance values indicated moderate differentiation between Q1 and Q2 (Supplemental Table S5), while both populations showed greater divergence from the admixed group.

### Linkage disequilibrium patterns and LD decay

A total of 4,307 linkage disequilibrium (LD) blocks were identified (Table 1), covering 675,441 kb, which corresponds to approximately 61.4% of the soybean genome. Genome-wide LD decay occurred at approximately 1.2 Mb (r^2^ = 0.22) (Supplemental Fig. 4). Chromosome-specific LD decay varied substantially, ranging from 1.9 Mb on chromosome 5 to 37.3 Mb on chromosome 16, indicating heterogeneous recombination landscapes across the genome (data not shown).

**Table 1:** Distribution of SNPs and characteristics of linkage disequilibrium (LD) blocks across the 20 chromosomes of 95 soybean genotypes

Chr	Number of SNPs	Blocks in disequilibrium	Blocks length (Kb)	Number of SNPs in LD blocks	Average density SNPs/per block
1	2555	136	30596	2466	18.13
2	3468	244	35197	3357	13.75
3	2931	197	32563	2785	14.13
4	2844	144	34546	2767	19.21
5	2,691	158	22996	2609	16.51
6	3429	288	38334	3242	11.25
7	2478	204	31660	2362	11.57
8	3284	231	33616	3151	13.64
9	3603	222	35521	3490	15.72
10	2725	149	36799	2666	17.89
11	2582	160	31144	2526	15.78
12	2089	121	21307	2031	16.78
13	4037	316	33504	3846	12.17
14	2698	232	36364	2563	11.04
15	4081	250	43065	3961	15.84
16	2892	396	23196	2397	6.05
17	2995	163	34405	2916	17.88
18	4539	355	49741	4238	11.93
19	3114	145	43136	3058	21.08
20	2677	196	27743	2555	13.03
Total	**61.712**	**4.307**	**675.441**	58.986*	14.67*

### Detection of loci under selection

**Fig. 4:**
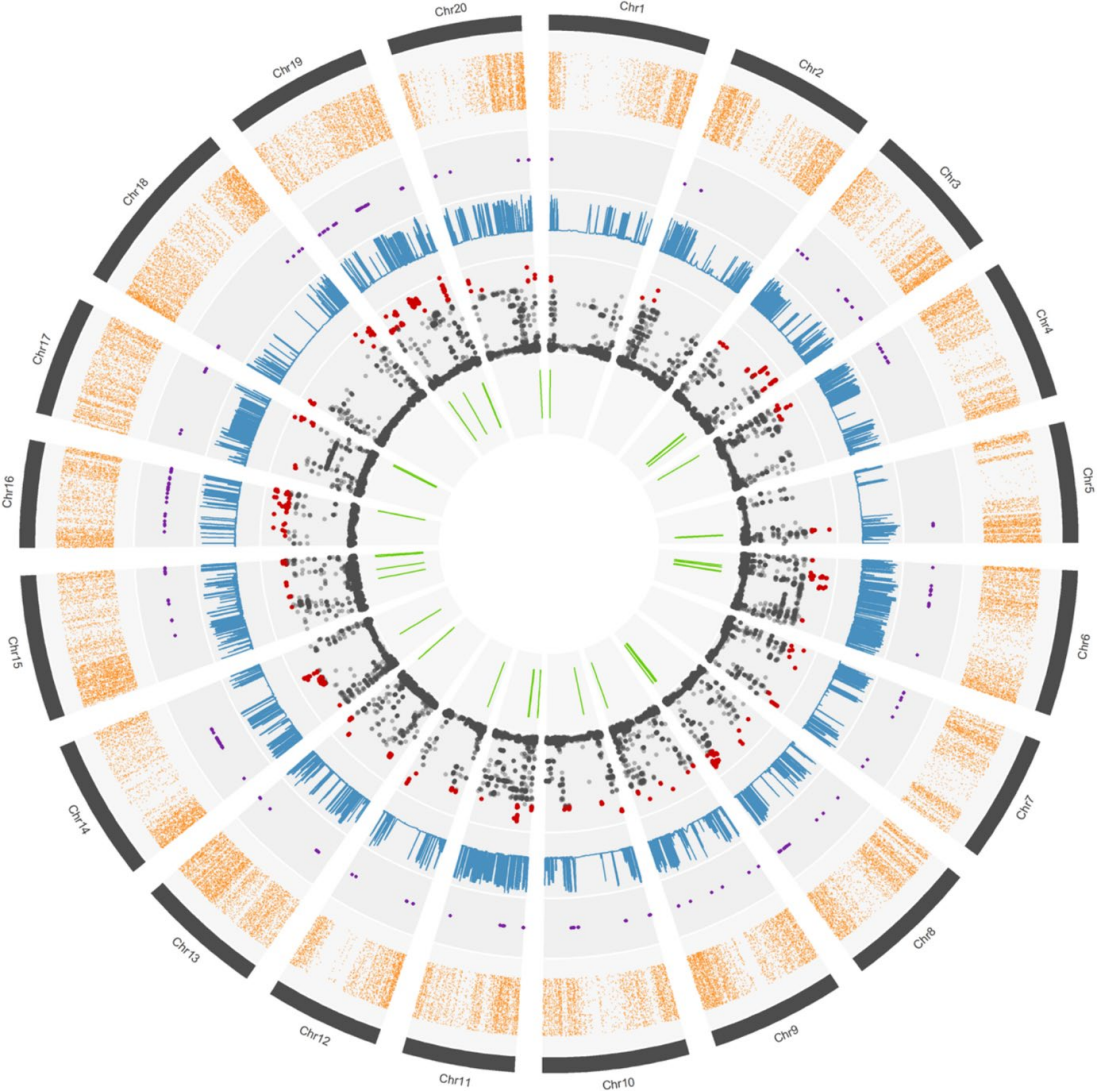
Circular representation of the genomic distribution of SNPs and selection signals across soybean chromosomes. The outermost circle (black) represents the 20 soybean chromosomes. Inner concentric circles follow the same chromosomal order and show, respectively, the distribution of the 61,712 total SNPs analyzed in this study (orange dots), SNPs under selection identified by BayeScan (purple dots), F_ST values (blue bars), and log₁₀(PO) values of SNPs, with red points indicating those considered significant. The innermost circle indicates the location of QTLs identified in regions under selection, obtained after linkage disequilibrium (LD) analysis (green bars)

**Fig. 5:**
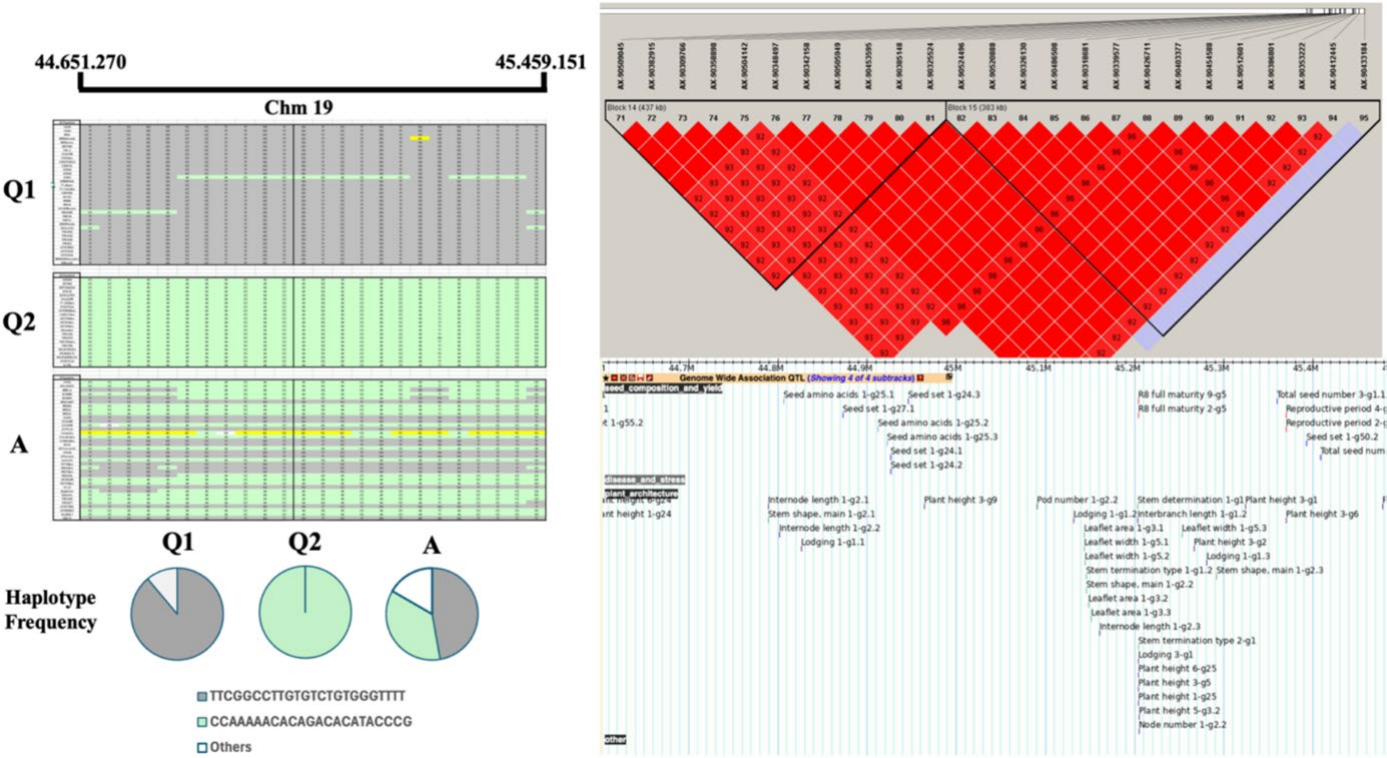
Haplotype structure and genomic region under selection on soybean chromosome 19. (**A**) Frequency of haplotypes identified in the region delimited by markers AX-90509045 and AX-90412445 across the ancestral (Q1), modern (Q2), and admixed (**A**) populations. (**B**) Haplotype blocks identified on chromosome 19 based on linkage disequilibrium analysis using Haploview software. (**C**) Corresponding genomic region visualized in SoyBase, highlighting previously reported QTLs located within the identified haplotype block

A total of 581 SNPs distributed across all 20 chromosomes were identified as candidates under selection (α > 0, as defined by BayeScan) between Q1 and Q2 populations (Fig. 4; Supplemental Table S6; Fig. 5). These SNPs overlapped with 102 previously reported QTLs associated with agronomically important traits (Supplemental Table S7; Supplemental Fig. 6). A prominent selection signal was detected on chromosome 19, spanning an interval of approximately 807 kb, where multiple SNPs exhibited strong differentiation between Q1 and Q2 populations.

To further characterize the SNPs identified under selection, we examined the genomic region on chromosome 19 delimited by markers AX-90509045 (44,651,270 bp) and AX-90412445 (45,459,151 bp) (Fig. 4; Supplemental Table S8). This region exhibited clear haplotype differentiation between populations Q1 and Q2, whereas admixed genotypes (population A) displayed a combination of alleles from both groups. Among the 36 genotypes assigned to population Q1, 32 shared an identical haplotype, with only four showing recombination with alleles predominant in Q2. In contrast, all 23 genotypes in population Q2 carried a single, uniform haplotype across this region. Within population A, 17 genotypes shared the Q2 haplotype, 13 shared the Q1 haplotype, and six exhibited recombinant haplotypes. Haplotype block analysis using the Solid Spine criterion identified two distinct blocks within this interval, both encompassing previously reported QTLs associated with agronomically important soybean traits (Fig. 5).

## Discussion

In this study, we characterized genome-wide diversity, population structure, and selection signatures in Brazilian soybean cultivars using high-density SNP data. Our results reveal a narrow genetic base, pronounced linkage disequilibrium, temporal population differentiation, and clear genomic signatures of selection, particularly distinguishing older and modern cultivars. These findings provide insights into how historical breeding strategies have shaped the contemporary Brazilian soybean genome.

Recent studies by Mendonça et al. (2022) and Silva et al. (2025) have provided important insights into the genetic diversity and selection patterns of Brazilian soybean. Building upon this foundation, our study extends previous efforts by incorporating both a higher marker density and a temporally structured germplasm panel, allowing a more detailed investigation of genomic changes across breeding eras. While Mendonça et al. (2022) analyzed 2,175 SNPs using GBS and Silva et al. (2025) employed the SoySNP50K array in a historical panel extending to 2016, the use of the higher density 180K Axiom array in our study (61,712 SNPs after filtering) enabled a finer resolution of haplotype structure, resulting in the identification of 4,307 haplotype blocks. This increased resolution is particularly relevant in soybean due to its extended linkage disequilibrium, which can obscure fine-scale genomic signals when marker density is limited.

In addition, our panel was specifically curated to represent distinct breeding periods while emphasizing modern elite cultivars that dominate current Brazilian agricultural systems. This design allows a more direct assessment of recent selection associated with contemporary practices, such as the widespread adoption of double cropping, and provides insight into how these pressures have reshaped haplotype structure in modern germplasm. Together, these results offer a refined and up-to-date perspective on the genomic consequences of soybean breeding, complementing previous studies by capturing recent selection dynamics that may not be fully represented in broader historical panels.

Genetic improvement relies on the collection, reorganization, and selection of genetic diversity, yet domestication and modern breeding often result in reduced variability (Louwaars 2018). Soybean, a predominantly self-pollinating species, is especially prone to high LD and limited genetic diversity (Yan et al. 2021). The extensive LD observed in this study (~ 1.2 Mb on average, with some regions showing substantially extended LD) contrasts with the shorter decay distances reported in North American elite germplasm (~ 574 kb) and landraces (10–500 kb) (Hyten et al. 2007; Song et al. 2015). This discrepancy likely reflects differences in the germplasm analyzed, as our panel represents elite Brazilian cultivars with a relatively narrow genetic base shaped by strong selection and breeding bottlenecks. For downstream analyses such as GWAS, this extensive LD may reduce fine-mapping resolution but can facilitate the detection of genomic regions associated with major traits due to the aggregation of signals within large haplotype blocks, as observed in other high-LD populations (Yan et al. 2021; Aleem et al. 2024). Fine-mapping limitations can be partially mitigated using haplotype-based approaches (Contreras-Soto et al. 2017). In addition, extended LD may be advantageous for genomic prediction, as lower marker densities can effectively capture the genetic variance of large haplotypic segments. However, this also highlights a trade-off between predictive ability and mapping resolution, reinforcing the importance of aligning analytical strategies with specific breeding and research objectives.

Soybean expansion in Brazil during the 1960 s relied heavily on U.S. germplasm, followed by crossing with local varieties and intensive selection for tropical adaptation (Bonato and Bonato 2002, Hiromoto and Vello 1986). The rapid expansion of soybean cultivation across different latitudes and environments required strong selection for early maturity, yield stability, and disease resistance. More recently, this selection process has been narrowed by the planting of second-crop corn, which demanded the development of earlier-maturing varieties while maintaining productivity. Our population structure results reflect this history, showing a clear separation between older cultivars (Q1) and modern elite materials (Q2) (Figs. 2 and 3). Despite contributions from multiple breeding programs, the high similarity among cultivars highlights the restricted diversity underlying Brazilian soybean breeding (Supplemental Fig. 2).

The reduced genetic diversity observed in population Q2 (Supplemental Table S4 and Supplemental Table S5), combined with its temporal expansion (Fig. 2 and Supplemental Fig. 3), strongly suggests directional selection associated with modern breeding goals. Traits such as early maturity, high productivity, and improved plant architecture have likely driven the genomic differentiation between Q1 and Q2 (Fig. 5).

Despite the relatively limited sample size, population Q2 includes several cultivars that are highly representative of current Brazilian soybean production. The cultivar Desafio RR, released in 2011, remains widely cultivated in the Brazilian Midwest, while more recent cultivars such as Bônus IPRO were extensively adopted and subsequently replaced by Olimpo IPRO, which currently occupies a substantial proportion of the national soybean area. These cultivars, developed by the GDM breeding program, exemplify the modern soybean ideotype in Brazil, characterized by early maturity, high yield potential, and indeterminate growth habit. The prominence of these materials in population Q2 supports the relevance of our genomic analyses for understanding the genetic composition of elite cultivars currently shaping Brazilian soybean production systems.

The identification of 581 SNPs under positive directional selection indicates widespread genomic differentiation between populations (Supplemental Fig. 5). While BayeScan is effective for detecting loci with elevated population differentiation, its mathematical framework has limited power to detect purifying selection in biallelic SNP datasets comparing only two populations (Foll and Gaggiotti 2008). Additionally, the extensive linkage disequilibrium in elite soybean may lead to outlier signals reflecting neutral hitchhiking. To enhance biological interpretation, we integrated these statistical signals with haplotype block boundaries and previously reported QTLs, focusing on genomic regions likely shaped by modern breeding.

The apparent discrepancy between the high number of SNPs under positive directional selection (581) and the relatively low number of overlapping previously reported QTLs (102) is a direct consequence of the extensive linkage disequilibrium (LD) in the elite soybean genome (Hyten et al. 2007; Song et al. 2015). Our analysis revealed that these significant SNPs are highly clustered within large haplotype blocks; consequently, multiple SNPs often tag the same genomic region, which explains the lower absolute count of unique annotated QTLs. This clustering of artificial selection signals driven by genetic linkage and high LD is a well-documented phenomenon in modern elite soybean populations (Wang et al. 2016; Yan et al. 2021). Interestingly, several haplotype blocks under selection did not overlap with any known QTLs in the SoyBase database. While this may partially reflect database limitations, it strongly suggests that modern Brazilian breeding might be fixing potentially novel or less-characterized genomic regions, which represent promising targets for future functional validation.

In the context of soybean improvement, these selection signatures represent the genomic footprints of selective sweeps and allele fixation driven by breeders. Among these, the region detected on chromosome 19 stands out as a major hotspot of selection. This region harbors multiple QTLs related to yield, seed composition, lodging resistance, and plant architecture, including the well-characterized Dt1 locus, a key regulator of stem growth habit (Fang et al. 2017; Zhang et al. 2015; Contreras-Soto et al. 2017; Zhou et al. 2015; Vuong et al 2015). The convergence of multiple selection signals in this interval suggests that chromosome 19 has played a central role in shaping modern Brazilian soybean cultivars.

Several reports have implicated this region in the control of yield-related and architectural traits, particularly through the Dt1 locus (Fang et al. 2017; Zhang et al. 2015; Contreras-Soto et al. 2017; Zhou et al. 2015; Vuong et al. 2015). Beyond growth habit, variation at Dt1 has been associated with multiple yield components, including branch density, node number, pod number, and total seed production, underscoring its pleiotropic effects on soybean performance.

Chromosome 19 harbors a high density of genes and QTLs relevant to soybean improvement, particularly within the interval between Glyma.19g188000 and Glyma.19g197400. This region includes candidate genes such as CS1 (Wang et al. 2025), involved in stem strength, auxin transport, and lodging resistance; GRF4 and ZPR3 homologs associated with leaf development (Wang et al. 2024); and genes implicated in seed size and weight, including a cell wall invertase (Glyma.19g195400) and a bZIP transcription factor (Glyma.19g193400) (Song et al. 2023; Zhang et al. 2025).

The robustness of our candidate loci is supported by substantial overlap with recent studies. We observed positional convergence with Mendonça et al. (2022) on chromosomes 6 (10.0 Mb), 18 (56.7 Mb), and 19 (44.0–45.5 Mb), where selection signals were particularly pronounced. In addition, our functional annotations are consistent with Silva et al. (2025), independently identifying regions associated with disease resistance, including Soybean Cyst Nematode (chromosomes 8, 11, 16, and 18) and Sclerotinia (chromosome 11), as well as traits related to flowering time (chromosomes 5, 6, 11, and 15), lodging (chromosome 19), and seed quality (chromosome 14). This convergence across different datasets and analytical approaches supports the relevance of these regions as key targets of selection in modern Brazilian soybean breeding.

In addition, several stable QTLs for key agronomic traits have been mapped to this interval on chromosome 19. These include major loci controlling 100-seed weight, such as qHSW-19-4 (Zhang et al. 2026), precisely mapped to the 44.84–44.85 Mb region, as well as qSw-19-1 and qSw-19-5 (Xu et al. 2023), which have shown consistent effects across multiple environments. The region also harbors qISO19-1 (Li et al. 2022), a novel locus influencing isoflavone content, including daidzein, genistein, and total isoflavones. Moreover, the qSS19-2 QTL (Wang et al. 2025), located approximately between 44 and 46 Mb, has been associated with stem strength and lodging resistance, with favorable alleles widely exploited in elite cultivars to reduce plant collapse.

While the major genomic region on chromosome 19 exhibits profound pleiotropic effects, our genome-wide haplotype analysis revealed that adaptation to the modern double-cropping system was a highly polygenic effort. Specifically, we identified multiple regions exhibiting signatures of positive directional selection (α > 0, BayeScan) overlapping with previously mapped QTLs for First flower, Reproductive period, and crucially, R8 full maturity distributed across several chromosomes (e.g., Gm04, Gm05, Gm08, Gm11, Gm15, and Gm16) (Supplemental Table S7). The direct identification of selection signatures within these specific maturity loci strongly corroborates that the demand for early-maturing varieties, driven by the second-crop corn succession system, has intensively reshaped the genomic landscape of modern Brazilian soybean. Furthermore, modifying the onset of flowering is often physiologically coupled with changes in reproductive duration (Zuo et al. 2013). By co-selecting these loci, breeders likely optimized a shortened vegetative cycle while maintaining an adequate seed-filling duration (Zhang et al. 2015). To ensure that this drastic phenological shift did not compromise productivity, direct selection for yield was concurrently applied independent of the Dt1 locus. Notably, selected loci on Gm06 (encompassing the 13.1 to 15.0 Mb region) co-localize precisely with major seed weight QTLs previously mapped through high-density GWAS (Zhang et al. 2016), underscoring the breeders' success in steadily increasing yield potential.

Beyond phenological and yield adaptations, modern breeding has also left strong selection signatures related to biotic stress resistance, most notably against the soybean cyst nematode (SCN, Heterodera glycines). SCN is a highly destructive pathogen that can cause yield losses of over 30%, making host plant resistance the most economically feasible strategy (Wendimu 2022). The genetic basis of SCN resistance is complex, requiring the introgression of multiple QTLs to achieve durable field resistance (Li et al. 2016). Reflecting this complexity, we detected significant signatures of positive directional selection (α > 0, BayeScan) acting on six distinct haplotype blocks overlapping with SCN resistance QTLs across six chromosomes (Gm03, Gm08, Gm15, Gm16, Gm19, and Gm20). The widespread fixation of these diverse SCN-related regions in the modern germplasm (Q2) illustrates the intense selective pressure applied to counteract this severe biological threat across Brazilian agricultural frontiers.

The genomic regions identified in this study provide valuable targets for marker-assisted selection and genomic selection. Understanding the genetic basis of divergence between historical and modern cultivars can guide breeding strategies aimed at balancing genetic gain with the maintenance of diversity. Introducing novel alleles from underutilized germplasm while preserving elite genomic backgrounds may be critical for sustaining long-term genetic improvement in Brazilian soybean.

Overall, our results demonstrate that modern Brazilian soybean cultivars are the product of intense selection acting on a narrow genetic base, resulting in pronounced genomic signatures of divergence. The identification of key genomic regions under selection, particularly on chromosome 19, provides a foundation for future breeding efforts aimed at improving productivity while mitigating further losses of genetic diversity.

## Supplementary Information

Below is the link to the electronic supplementary material.

Supplementary file1 (DOCX 4458 KB)

Supplementary file2 **Detailed results of diversity, population structure, and selection analyses in Brazilian soybean genotypes. **The files include genetic distance matrices, genotype information, AMOVA results, estimates of genetic diversity and differentiation, complete genotyping data, QTLs located in regions under selection, and haplotype blocks identified on chromosome 19. (XLSX 332 KB)

## Data Availability

The datasets generated are available from the corresponding author on reasonable request.
