# Supplementary material for: Genome-wide signatures of selection and genetic diversity reveal the impact of modern breeding on Brazilian soybean
Source: Mol Breed. 2026 May 28;46(6):58. doi: 10.1007/s11032-026-01679-0 (PMC13219583; doi:10.1007/s11032-026-01679-0)
Supplement: Supplementary file 1 — Supplementary file1 (DOCX 4458 KB) [file 11032_2026_1679_MOESM1_ESM.docx]

**Supplemental Figures S1 to S6**

**Genome-wide signatures of selection and genetic diversity reveal the impact of modern breeding on Brazilian soybean**

Brizza Fernandes dos Santos Vargas; Newton Deniz Piovesan; Cleberson Ribeiro, Maximiller Dal-Bianco


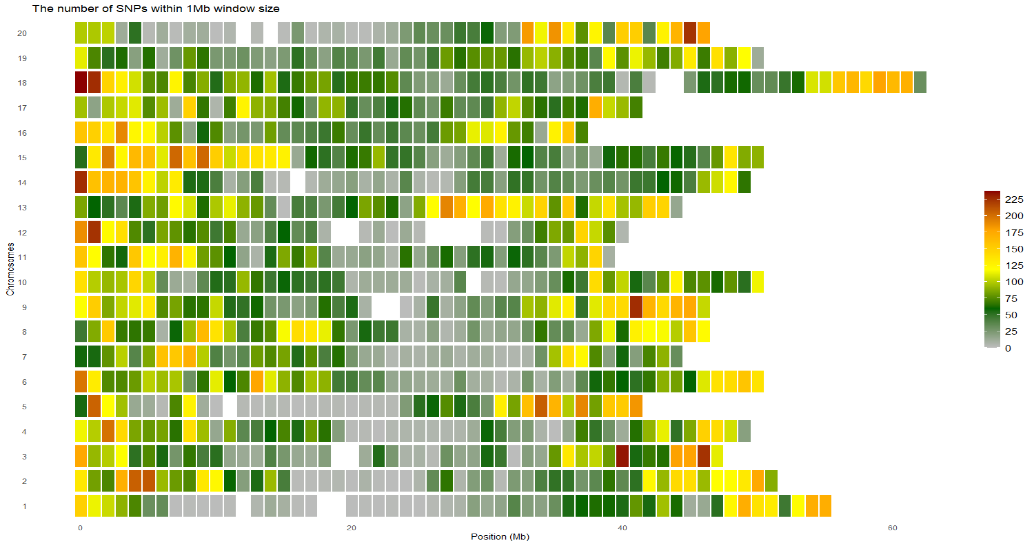


**Supplementary Figure S1. SNP density along soybean chromosomes.** Density and distribution of SNPs across the 20 soybean chromosomes, estimated using sliding windows of 1 Mb. The horizontal axis represents physical distance in megabases (Mb), while colors indicate variation in SNP density along each chromosome.


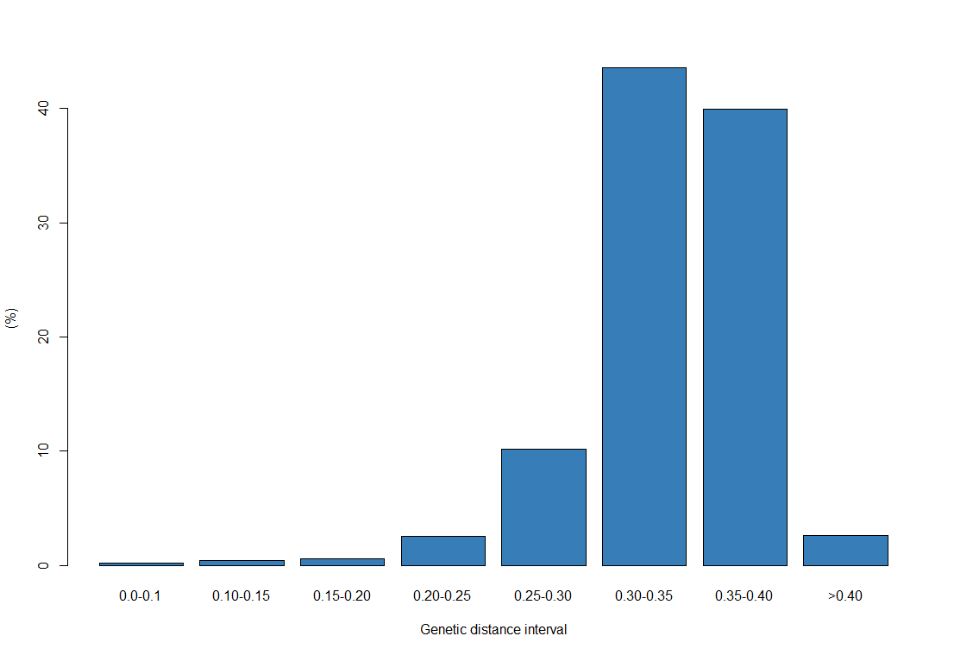


**Supplementary Figure S2. Frequency distribution of genetic distances among soybean genotypes.** Frequency distribution of genetic distances estimated among 95 soybean genotypes based on 61,712 SNP markers, using the genetic distance matrix proposed by Endelman and Jannink (2012). The x-axis represents genetic distance intervals, while the y-axis indicates the percentage of occurrence for each interval.

.
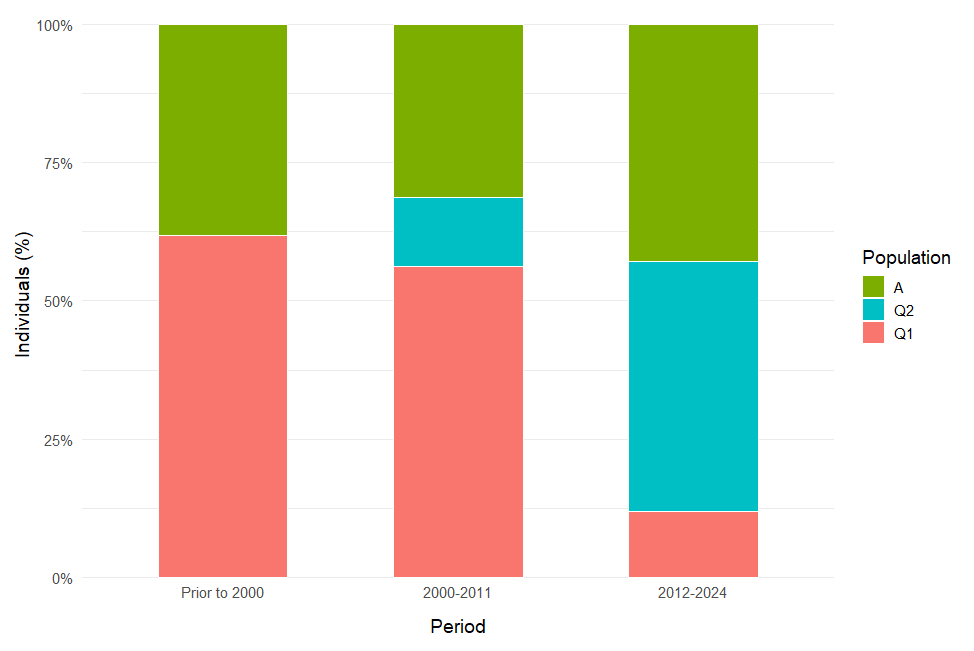


**Supplementary Figure S3. Proportion of soybean genotypes by genetic population over time.** The bar chart shows the percentage distribution of soybean genotypes grouped by release period. Colors indicate the genetic populations inferred from the population structure analysis (K = 2), where Q1 represents the ancestral/older population, Q2 represents the modern population, and A represents the admixed group.


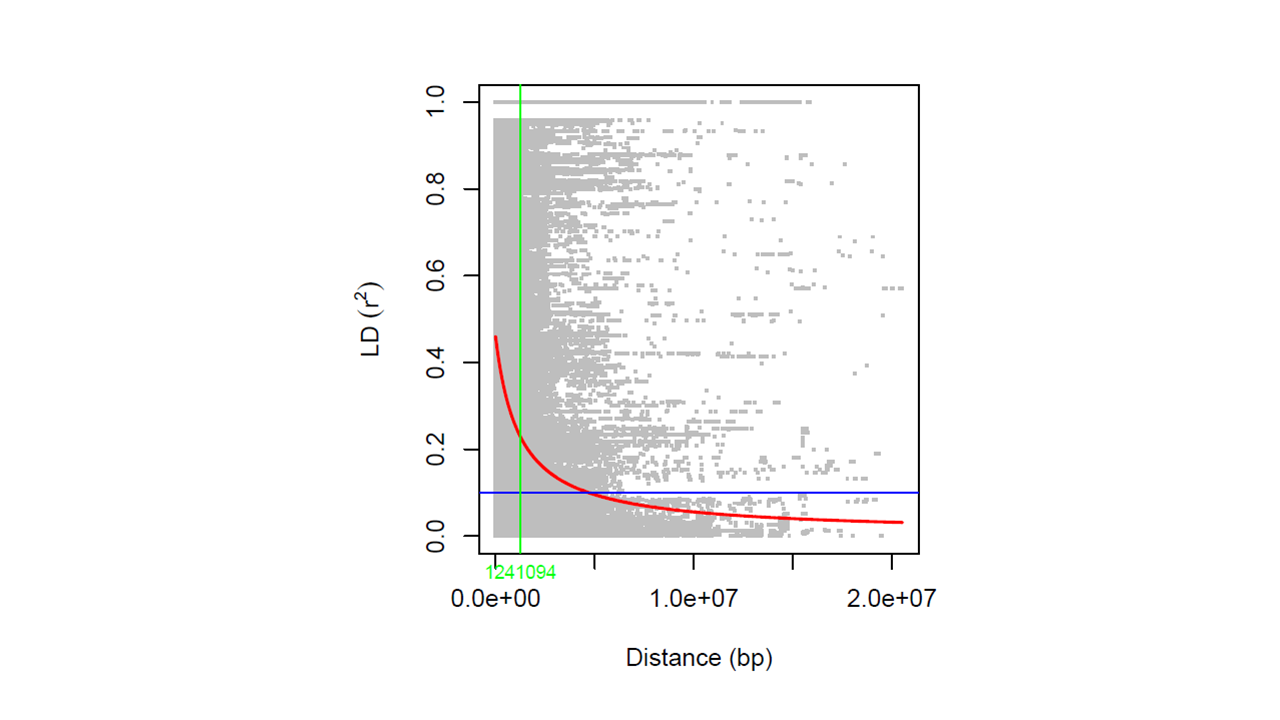


**Supplementary Figure S4. Linkage disequilibrium (LD) decay.** The y-axis represents mean LD (r²) values, and the x-axis represents the physical distance between SNP pairs (bp). The red line indicates the nonlinear regression of r² as a function of distance. The blue line marks the r² = 0.1 threshold, used as a reference for low marker correlation. The green line indicates the LD half-decay distance, estimated at approximately 1.2 Mb.


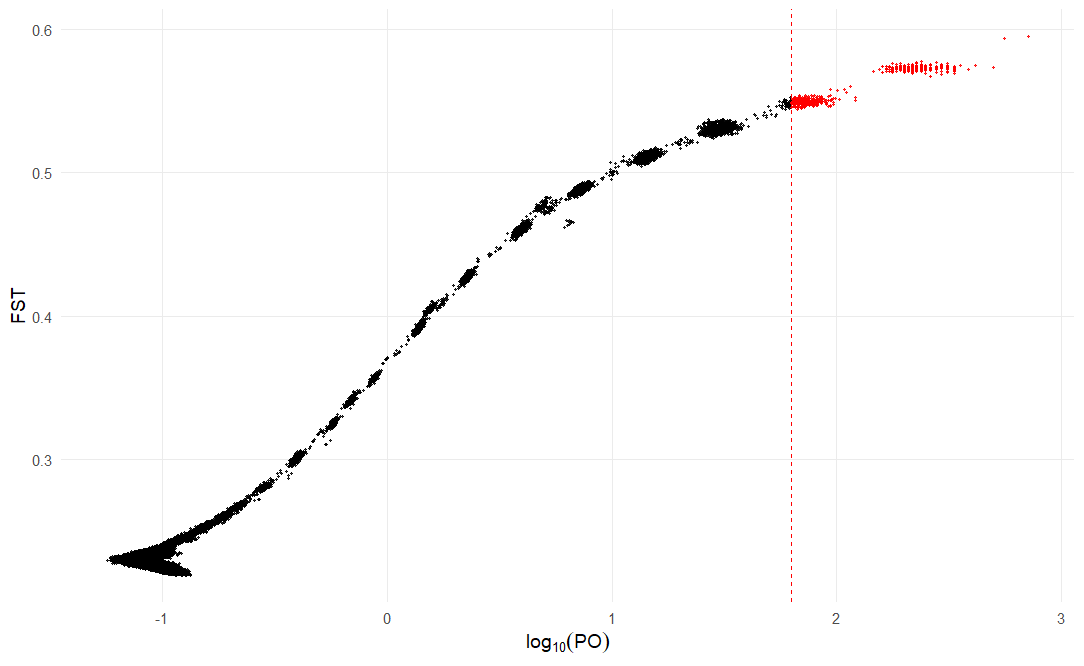


**Supplementary Figure S5. Identification of candidate loci under selection using BayeScan.** The plot shows the relationship between log₁₀(Posterior Odds) (x-axis) and F_ST per locus (y-axis). Black dots represent all SNPs analyzed. Red dots indicate candidate loci under diversifying selection (α ≥ 0) with q-value ≤ 0.01 (1% FDR). The red dashed vertical line indicates the log₁₀(PO) threshold corresponding to the significance criterion, defined as the lowest log₁₀(PO) value among loci with q-value ≤ 0.01, separating neutral loci from those showing evidence of selection.


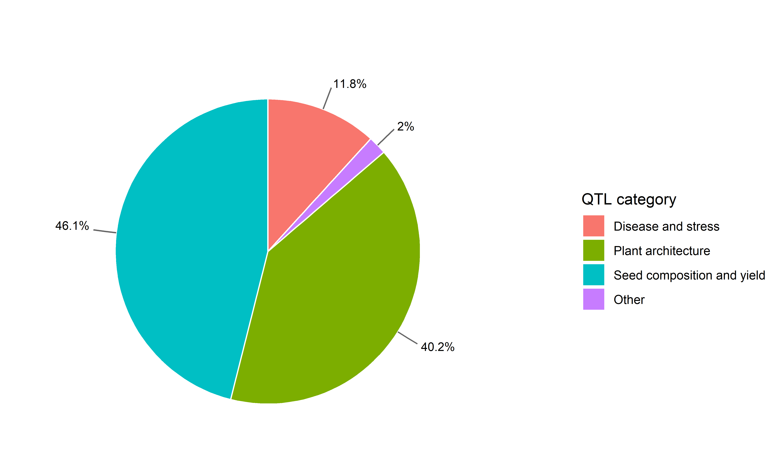


**Supplementary Figure S6. Distribution of previously reported QTLs in genomic regions under selection.** The pie chart shows the proportion of the 102 previously reported QTLs associated with agronomically important traits that were identified within genomic regions under selection. QTL information was obtained from the SoyBase database.
